# Metastatic malignant melanoma presenting as bilateral acute mastitis in a lactating woman: a case report and literature review

**DOI:** 10.3389/fonc.2026.1892580

**Published:** 2026-07-06

**Authors:** Zilin Wang, Shaowu Zeng, Qiaorong Zhou, Jujiang Guo

**Affiliations:** 1 Department of Breast Diseases, Department of Obstetrics and Gynecology, Women and Children’s Hospital, School of Medicine, Xiamen University, Xiamen, China; 2 Department of Pathology, Department of Obstetrics and Gynecology, Women and Children’s Hospital, School of Medicine, Xiamen University, Xiamen, China

**Keywords:** acute mastitis, breast metastasis, lactation, malignant melanoma, triple-negative mimic

## Abstract

Malignant melanoma, an aggressive neoplasm with high metastatic potential, rarely metastasizes to the breast, with bilateral involvement being exceptionally uncommon. This case report details a 36-year-old postpartum woman who presented with bilateral, symmetrical, rock-hard breast swelling and erythema, initially misdiagnosed as severe lactational mastitis. Comprehensive evaluation ultimately revealed diffusely metastatic malignant melanoma. The primary site was identified as a previously excised cutaneous lesion on the chest wall, originally reported as a benign compound nevus. The patient exhibited a rapidly progressive and refractory disease course, failing to respond to sequential targeted therapy, immunotherapy, and chemotherapy, and succumbed to the disease within seven months of diagnosis. This case underscores a profound diagnostic pitfall, highlighting the potential for metastatic melanoma to mimic benign inflammatory breast conditions, particularly in the physiologically altered postpartum period. It emphasizes the critical need for a high index of suspicion for metastatic disease in atypical breast presentations and reinforces the imperative of meticulous history-taking, including a thorough review of all prior skin pathology, in patients with malignancy of unknown origin. The aggressive clinical course further illustrates the formidable therapeutic challenges in such advanced presentations of metastatic melanoma.

## Introduction

Malignant melanoma, an aggressive neoplasm arising from melanocytes, is characterized by its high metastatic potential and increasing global incidence (1). While common sites of distant spread include the lungs, brain, and liver, metastasis to the breast is an exceptionally rare event, accounting for only 1.3% to 2.7% of reported metastatic cases (2). Breast metastases from extramammary malignancies themselves are uncommon, representing approximately 0.5% to 2% of all malignant breast tumors, with melanoma being one of the most frequent primary sources alongside carcinomas of the lung and gastrointestinal tract (3, 4). Clinically, such metastases typically present as solitary, palpable breast masses, often mimicking primary breast carcinoma on imaging, which can lead to diagnostic pitfalls and inappropriate management (2, 5).

The clinical presentation of metastatic melanoma to the breast is usually unilateral and focal. Cases of bilateral breast involvement are exceedingly rare and typically occur in the context of widely disseminated, advanced-stage disease with a known primary cutaneous lesion (6). The diagnostic challenge is further compounded when breast metastasis serves as the initial manifestation of an otherwise occult primary melanoma, a scenario that is poorly documented in the literature (7). Moreover, the occurrence of such an event in the specific physiological context of the postpartum, lactating breast adds a profound layer of diagnostic complexity. The normal physiological engorgement, increased vascularity, and potential for inflammatory conditions like mastitis in this period can perfectly mask underlying malignant pathology, leading to critical delays in diagnosis.

This case report presents a uniquely challenging and instructive clinical scenario: a 36-year-old postpartum woman presenting with bilateral, symmetrical, rock-hard breast swelling and erythema, initially suspected to be severe lactational mastitis or abscess. The subsequent revelation of diffusely metastatic malignant melanoma originating from a previously excised and histologically benign-appearing cutaneous nevus underscores several critical and underappreciated clinical dilemmas. It highlights the potential for malignant melanoma to masquerade as a benign inflammatory breast condition, the possibility of bilateral breast parenchyma serving as the first site of detectable metastasis from an occult primary, and the ominous implication of a historically “benign” skin lesion. The aggressive course and poor response to sequential targeted therapy, immunotherapy, and chemotherapy further illustrate the formidable therapeutic challenges in such advanced presentations. Therefore, this case holds significant clinical value for publication, as it elucidates an extreme diagnostic pitfall, emphasizes the necessity of a high index of suspicion for metastatic disease in atypical breast presentations, and reinforces the imperative for meticulous history-taking and comprehensive skin examination in patients with metastatic malignancy of unknown origin.

## Case presentation

### Patient information

**Figure 1:**
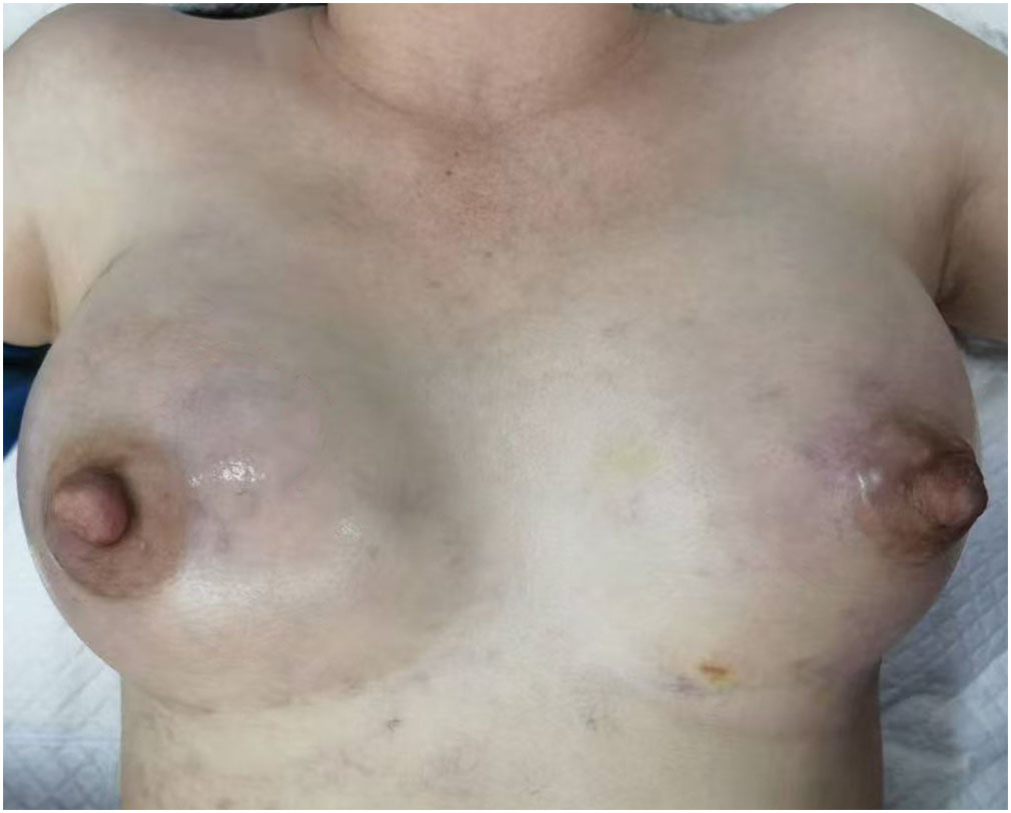
Clinical presentation of the patient on admission. Bilateral symmetrical severe breast swelling with tense, shiny skin and prominent superficial vascular dilation, mimicking acute lactational mastitis.

A 36-year-old female patient presented to Xiamen Maternal and Child Health Hospital on May 14, 2025, with a chief complaint of bilateral breast distension and pain for two weeks. Her medical history was notable for two cesarean sections, performed 7 years and 50 days prior, respectively, with unremarkable postoperative recovery. She had no other underlying medical conditions. Her family history included the death of her father from lung cancer, with no other known family history of malignancy. Upon admission, physical examination showed bilateral symmetrical severe breast swelling with tense, shiny skin and prominent superficial vascular dilation (**Figure 1**). Mild erythema was noted, with no skin dimpling, nipple retraction, inversion or erosion. The bilateral breasts were stony hard and tender on palpation. A small amount of milky discharge was observed upon nipple compression. No enlarged axillary or supraclavicular lymph nodes were palpated, and only normal axillary glandular tissue was detected bilaterally. Ultrasonography demonstrated findings consistent with lactational changes (BI-RADS 1), a solid nodule in the right breast (BI-RADS 3), scattered cystic areas bilaterally (BI-RADS 3), and left and right accessory breast tissue with a cystic nodule (BI-RADS 2).

### Clinical findings

All routine laboratory tests, including blood tests, inflammatory markers, coagulation function, biochemistry and infectious disease serology, were within normal ranges. Acute lactational mastitis with suspected abscess was highly considered, and the patient received emergency general anesthesia surgery on the day of admission labor. Intraoperatively, incision of the skin and subcutaneous tissue revealed sanguineous fluid but no purulent material. Ultrasound-guided dissection of the presumed cystic cavities also failed to identify pus. The surrounding breast tissue was noted to be firm, raising suspicion for a neoplastic process. Therefore, a vacuum-assisted core needle biopsy was performed on both breasts. Intraoperative frozen section analysis of specimens from both the left and right breasts showed tumor cells arranged in solid sheets with eosinophilic cytoplasm, eccentric nuclei, and atypia, suggesting a malignant neoplasm.

### Diagnostic assessment

A subsequent PET/CT scan revealed the following findings: 1. Bilateral breast enlargement with multiple nodular lesions and diffusely increased metabolic activity, consistent with a malignant breast neoplasm based on the pathological findings. 2. Multiple enlarged lymph nodes in the right supraclavicular region, bilateral anterior chest wall intermuscular spaces, and bilateral axillae with varying degrees of increased metabolic activity, considered metastatic. 3. Multiple pulmonary nodules and masses with increased metabolism, lymphangitic carcinomatosis, and irregular pleural thickening with mild hypermetabolism, all suggestive of metastasis, accompanied by small bilateral pleural effusions. 4. Multiple slightly hypodense hepatic nodules with varying degrees of increased metabolism, and a subcutaneous soft tissue nodule in the mid-upper abdominal wall with mild hypermetabolism, also considered metastatic. 5. Multiple foci of heterogeneous increased bone density throughout the skeleton with varying metabolic activity, consistent with osseous metastases.

**Figure 2:**
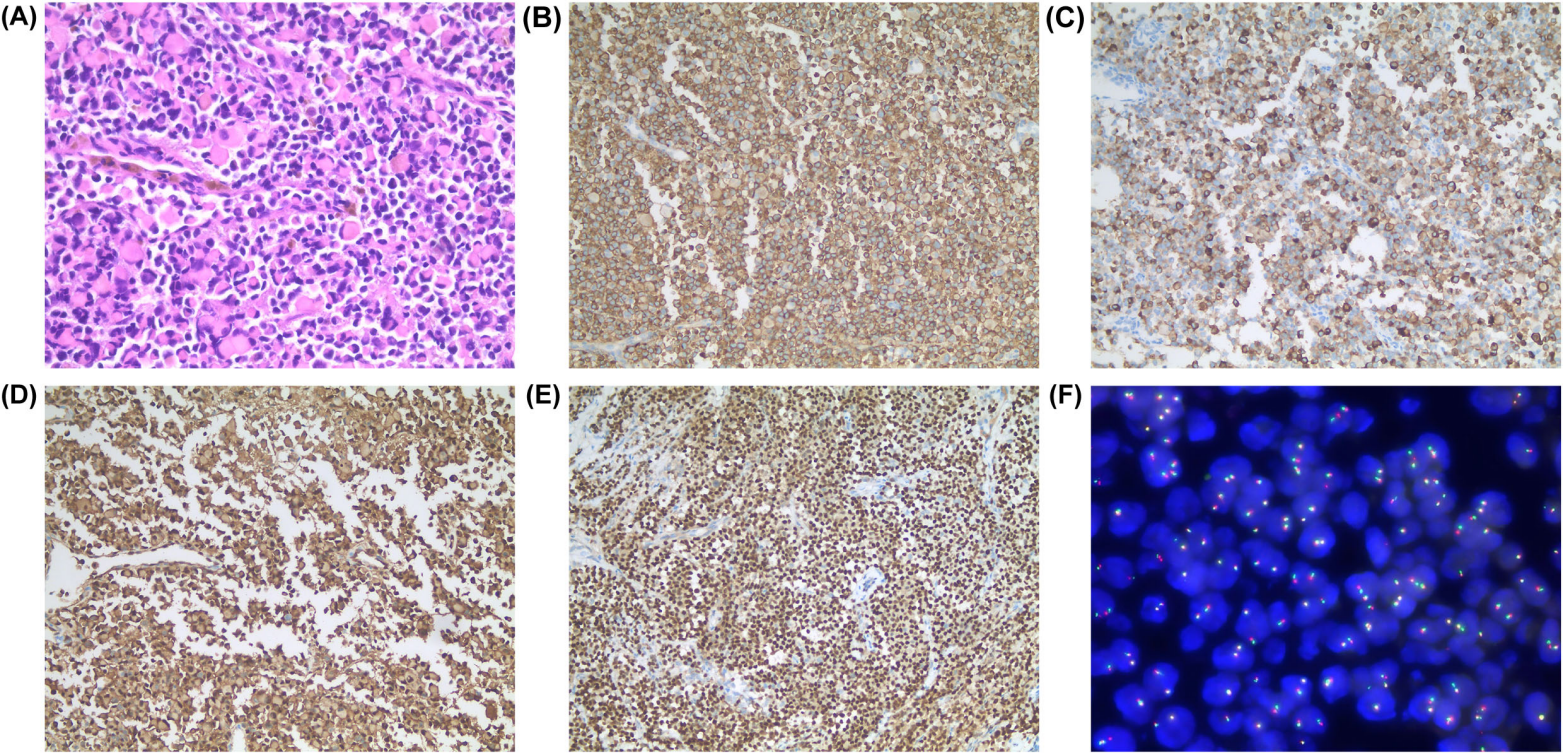
Histopathological and molecular findings of breast lesions. **(A)** Hematoxylin and eosin (HE) staining reveals infiltrative malignant tumor cells. **(B–E)** Immunohistochemical results: **(B)** HMB-45 positive; **(C)** Melan-A positive; **(D)** S-100 positive; **(E)** SOX10 positive. **(F)** Fluorescence *in situ* hybridization (FISH) demonstrates negative EWSR1 gene rearrangement.

Final pathology from the microinvasive biopsy specimens of both breasts demonstrated tumor cells arranged in solid sheets with infiltrative growth. The cells were small and uniform, with eosinophilic cytoplasm, eccentric, atypical nuclei, visible pigment, and mitotic figures. Immunohistochemical staining and genetic testing confirmed the diagnosis of malignant melanoma. Vascular invasion was noted in multiple vessels. The immunohistochemical profile was as follows (**Figures 2B–E**): CK-pan (–), Vimentin (+), S-100 (+), HMB-45 (+), MelanA (+), SOX10 (+), MUM1 (weak +), LCA (-), TTF-1 (-), ER (-), PR (-), HER-2 (0), Ki-67 (55%+), p53 (wild-type expression +), CK5/6 (-), p63 (-), E-Ca (+), P120 (membranous +), AR (-), GATA3 (-), TRPS1 (weak +), CD38 (-), CD138 (-), desmin (partial +), MyoD1 (-), Myogenin (-), calretinin (-), INSM-1 (-), INI-1 (+, normal expression), BRG1 (+, normal expression), MPO (-), CD30 (-). Fluorescence *in situ* hybridization (FISH) for the EWSR1 gene rearrangement was negative (**Figure 2F**). Molecular testing identified a BRAF V600E mutation.

**Figure 3:**
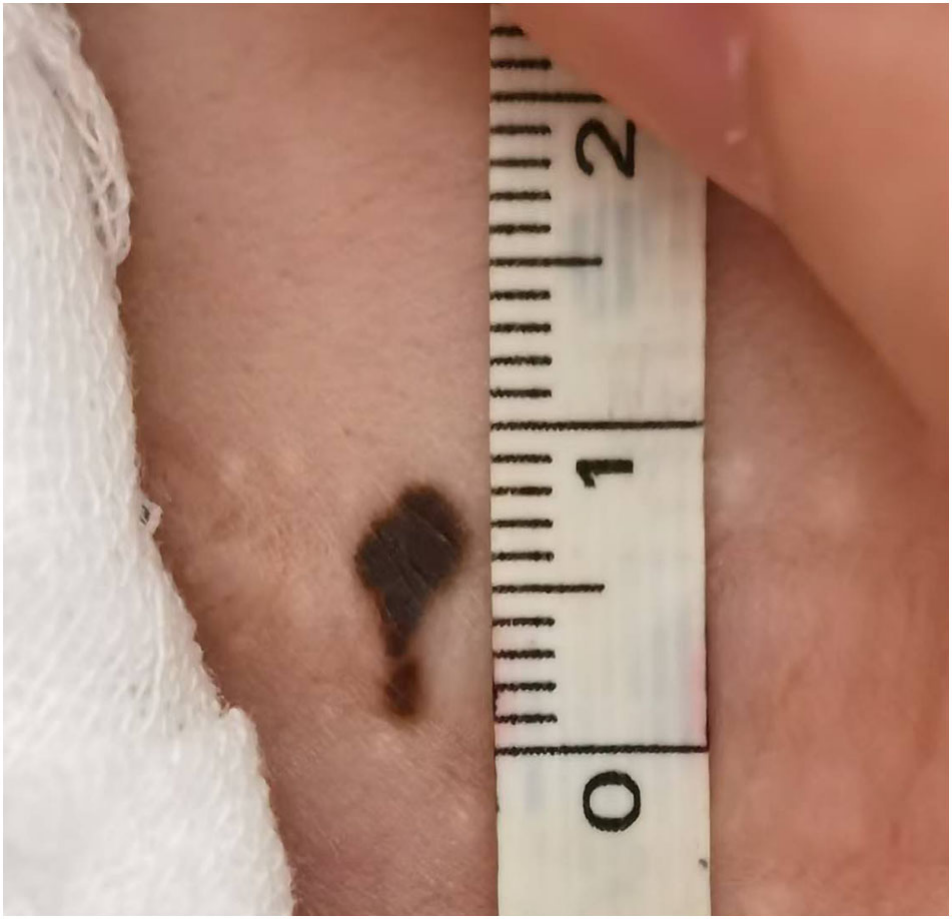
Suspected primary cutaneous melanocytic lesion on the right chest wall. An irregular, asymmetric, 0.8-cm pigmented plaque with variegated coloration on the right anterior chest wall near the anterior axillary line, which was clinically suspected as the primary site of malignant melanoma.

A thorough dermatological examination identified an irregular, asymmetric, 0.8-cm pigmented plaque with variegated coloration on the right anterior chest wall near the anterior axillary line (**Figure 3**). The patient recalled undergoing excisional biopsy of a “mole” at this exact location at an outside hospital four years prior (2021), which was originally reported as a “compound nevus with active nevus cell proliferation” with clear margins. No further treatment or surveillance was recommended. In 2025, archived slides were submitted for expert consultation at Fujian Cancer Hospital, which reclassified the lesion as a “melanocytic neoplasm with cellular atypia” with recommended FISH testing. Immunohistochemistry demonstrated positivity for Melan-A, HMB-45, S-100, SOX10, tyrosinase, and BRAF, with partial CD117 expression and CyclinD1 overexpression (80%). Notably, p16 was lost, the Ki-67 index was 15%, and PHH3 showed 5 mitoses/mm²—features atypical for a benign nevus. PRAME was negative. These findings raised concern for an atypical melanocytic proliferation of uncertain malignant potential; however, definitive classification as melanoma was not established due to absent FISH studies. Given the current widespread metastatic melanoma and the temporal and anatomical correlation, the 2021 chest wall lesion is highly suspected to represent the primary cutaneous melanoma or its precursor, with the breast disease likely constituting metastatic recurrence after a four-year interval.

### Treatment course and outcome

The patient was subsequently transferred to another institution. On May 28, 2025, she commenced targeted therapy with dabrafenib (150 mg twice daily) and trametinib (2 mg once daily). After more than four months of treatment, a restaging evaluation on September 25, 2025, demonstrated disease progression, including progression of pulmonary metastases and new metastases in the brain parenchyma and uterus. Consequently, pembrolizumab was added. Based on the patient’s genetic test results, physical condition and current clinical guidelines for metastatic melanoma, this targeted combination therapy was selected as the first-line regimen. On October 24, 2025, the regimen was changed to temozolomide (300 mg on days 1-5), cisplatin (40 mg on days 1-2, 30 mg on day 3), and bevacizumab (375 mg on day 1). However, the disease remained refractory to this treatment. Due to pulmonary lymphangitic carcinomatosis, the patient could not tolerate radiotherapy. On November 14, 2025, treatment with dabrafenib and trametinib was resumed. This was later switched to a combination of pembrolizumab and ipilimumab, which also proved largely ineffective.

Despite multiple lines of therapy, the disease demonstrated relentless progression. The patient developed worsening respiratory failure from pulmonary lymphangitic carcinomatosis, hepatic dysfunction, and neurological decline. She transitioned to comfort-focused care and died on December 31, 2025, approximately 7 months after the initial breast presentation.

## Discussion

Metastatic melanoma to the breast is a rare event, accounting for only 1.3% to 2.7% of all metastatic breast tumors, and its presentation as bilateral, diffuse involvement is exceedingly uncommon (1, 2). The majority of reported cases describe a solitary, palpable breast mass, often in patients with a known history of cutaneous melanoma, as demonstrated by a case of a 51-year-old woman with a prior back melanoma who presented with a single left breast mass (1), and another involving a 64-year-old woman with a history of excised skin melanoma presenting with a circumscribed mass (8). A review of 99 patients with melanoma identified in the breast parenchyma found that 56% had a history of prior skin melanoma, with the most common presentation being a solitary mass in the upper outer quadrant (9). Furthermore, bilateral breast metastases from melanoma are exceptionally rare, with only a few case reports, such as a 69-year-old woman with a history of cutaneous melanoma who developed bilateral breast masses (6), and a case of rectal melanoma metastasizing to both breasts (10). In stark contrast to these cases, our patient presented with bilateral, symmetrical, and diffuse breast involvement that clinically mimicked acute mastitis during the postpartum lactation period, a presentation not previously described. This case is also distinct because the primary lesion was an inconspicuous, previously resected “compound nevus” on the chest wall, discovered only after a thorough skin examination prompted by the breast pathology, rather than a known melanoma diagnosis (2). The diagnostic challenge was further compounded by the non-specific imaging findings, classified as BI-RADS 3, which are consistent with the literature describing melanoma metastases as largely non-specific, circumscribed masses (11).

The occurrence of this presentation in a 36-year-old woman during the postpartum period introduces a unique clinical scenario. While melanoma can metastasize to the breast in young women, as reported in a pregnant patient with a rapidly progressive course (12), our case is the first to describe bilateral, diffuse mammary involvement mimicking a benign inflammatory condition in the lactating breast. The physiological changes of lactation, including increased vascularity and lymphatic flow, may have contributed to the unusual pattern of diffuse, bilateral spread, potentially creating a favorable microenvironment for metastatic seeding. This presentation led to an initial misdiagnosis of acute mastitis, a common pitfall also highlighted in a case of primary breast melanoma presenting as a recurrent breast abscess (13). The aggressive nature of the disease was evident, with rapid progression despite targeted therapy with BRAF/MEK inhibitors and subsequent immunotherapy, leading to death within seven months of diagnosis. This poor prognosis aligns with the literature, which reports a median survival of only 11.5 months for patients with melanoma identified in the breast parenchyma (9) and reflects the grim outlook for metastatic melanoma, where early detection of the primary lesion is crucial for management (14, 15). This case underscores the necessity of considering metastatic melanoma in the differential diagnosis of atypical breast lesions, especially in patients with a history of any pigmented skin lesion, even those previously diagnosed as benign.

This case highlights several critical diagnostic pitfalls and offers insights into the underlying pathobiology of metastatic melanoma. The foremost diagnostic challenge was the profound clinical mimicry of acute mastitis, a common postpartum condition. The bilateral, diffuse, and painful breast enlargement with erythema, in the absence of systemic infection markers, led to a misdiagnosis and unnecessary surgical exploration. This scenario echoes other reports of melanoma mimicking benign inflammatory conditions, such as a case of primary breast melanoma presenting as a recurrent abscess (13) and an anorectal melanoma misdiagnosed as hemorrhoids (16). The non-specific imaging findings (BI-RADS 3) further contributed to the diagnostic delay, a known issue as melanoma metastases often appear as circumscribed masses without pathognomonic features (11). A second critical trap was the history of a previously resected “compound nevus” with “active nevus cells,” which was initially dismissed as benign. This case underscores the imperative for a meticulous review of all prior skin pathology, as a small primary lesion, especially an amelanotic or regressed one, can be easily overlooked (2). The discovery of the primary site only after a thorough skin examination prompted by breast pathology reinforces that a high index of suspicion is crucial for identifying occult primaries in patients presenting with metastases (17, 18).

From a mechanistic perspective, this case offers a unique window into the biology of melanoma dormancy and metastatic tropism. The interval of four years between the excision of the primary nevus and the development of widespread metastases is consistent with the phenomenon of tumor dormancy, where malignant cells can remain quiescent for extended periods before reactivation. The specific pattern of bilateral, diffuse breast involvement is exceptionally rare and may be explained by the unique microenvironment of the lactating breast. The profound physiological changes of lactation, including increased vascularity, lymphatic drainage, and hormonal stimulation (e.g., estrogen, progesterone, prolactin), could create a “fertile soil” for circulating melanoma cells, a concept supported by evidence that hormonal and immune alterations during pregnancy can aggravate melanoma prognosis (12). The rapid, aggressive clinical course and poor response to both targeted therapy (BRAF/MEK inhibitors) and immunotherapy (anti-PD-1/CTLA-4) suggest the presence of intrinsic or rapidly acquired resistance mechanisms. While the BRAF mutation status was not determined in this case, the lack of durable response to targeted therapy is consistent with the development of resistance through mechanisms such as MAPK pathway reactivation, PI3K/AKT pathway activation, or the emergence of a fibrotic stroma that promotes drug tolerance (19). This case demonstrates that the combination of an immunosuppressive postpartum state and the altered microenvironment of the lactating breast may synergistically facilitate both the reactivation of dormant melanoma cells and the rapid progression of therapy-resistant disease.

This case compels a critical reflection on several clinical and biological dimensions. First, the diagnostic odyssey underscores the paramount importance of maintaining a high index of suspicion for metastatic disease when confronted with atypical breast presentations, particularly in the postpartum period. The initial misdiagnosis as acute mastitis, despite the absence of infectious markers, highlights a dangerous cognitive bias where common conditions are favored over rare but catastrophic alternatives. The failure to immediately consider a metastatic process, even in the absence of a known primary, led to a delay in definitive diagnosis. This case mandates that any non-resolving or atypical breast lesion, especially in a patient with a history of any pigmented skin lesion, should prompt early consideration of metastasis and a low threshold for tissue biopsy. Furthermore, the discovery of the primary lesion only after a targeted skin examination reinforces the necessity of a meticulous and complete physical examination, including a thorough skin survey, in all patients with newly diagnosed metastatic malignancy of unknown origin. The inability to re-review the original pathology of the excised nevus represents a significant limitation, leaving the question of whether the lesion was truly benign or a misdiagnosed early melanoma unanswered. This highlights the critical need for clinicians to obtain and re-evaluate prior pathology when a patient’s clinical trajectory changes unexpectedly.

## Conclusion

In summary, this case report documents the first description of bilateral, diffuse breast metastases from an occult malignant melanoma masquerading as acute lactational mastitis. It serves as a stark reminder that malignant melanoma can present in a profoundly deceptive manner, with breast involvement as the first and only sign of systemic disease. The aggressive clinical course and poor response to multimodal therapy, resulting in death within seven months of diagnosis, underscore the formidable nature of this disease once it has disseminated. The unique physiological state of lactation may have created a permissive niche for both the reactivation of dormant melanoma cells and the rapid progression of therapy-resistant disease. While acknowledging the inherent limitations of a single case report, this presentation offers a critical educational lesson for clinicians across multiple specialties. It reinforces the essential principle that the differential diagnosis of breast pathology must include metastatic disease, even in young, postpartum women, and that a comprehensive history and physical examination remain indispensable tools in uncovering the underlying etiology of unusual clinical presentations.

## Data Availability

All clinical information involved in this case is confidential and cannot be publicly disclosed to protect patient privacy. Relevant de-identified clinical records can be requested from Zilin Wang via email: wangzlin9@alumni.sysu.edu.cn.
